# Characterization of *Aphanizomenon ovalisporum* amidinotransferase involved in cylindrospermopsin synthesis

**DOI:** 10.1002/mbo3.78

**Published:** 2013-03-26

**Authors:** Ángel Barón-Sola, Miguel A Gutiérrez-Villanueva, Francisca F del Campo, Soledad Sanz-Alférez

**Affiliations:** Departamento de Biología, Universidad Autónoma de MadridCampus de Cantoblanco, 28049, Madrid, Spain

**Keywords:** Amidinotransferase, cyanobacteria, cylindrospermopsin, enzyme activity, guanidinoacetate, toxin

## Abstract

An increasing abundance of *Aphanizomenon ovalisporum* in water bodies from diverse world regions has been reported in the last few years, with the majority of the isolated strains producing the toxin cylindrospermopsin (CYN), leading to a rise in ecological and health risks. The understanding of CYN synthesis is crucial in the control of CYN production. An amidinotransferase (AMDT) seems to be the first enzyme involved in the synthesis of CYN. In this study, we have cloned and overexpressed the *aoaA* gene from the constitutive CYN producer *A. ovalisporum* UAM-MAO. The recombinant purified AoaA was characterized, confirming that it is an l-arginine:glycine AMDT. It shows an optimal activity between 32 and 37°C, at pH from 8 to 9. The activity exhibits a mixed (ping-pong/sequential) kinetic mechanism, and is inhibited by the reaction product guanidine acetate (GAA) in a noncompetitive manner. Mg^2+^ stimulates AoaA activity while Co^2+^ and Mn^2+^ inhibit it. AoaA conserves the critical residues of the catalytic site and substrate specificity of AMDTs, as the previously reported AMDT from *Cylindrospermopsis raciborskii* Cyr. Both proteins can be included in a new group of prokaryotic AMDTs involved in CYN production.

## Introduction

*Aphanizomenon ovalisporum* is a filamentous cyanobacterium that in the last decade has become a cause for concern in fresh-water habitats, due to its ability to produce the potent alkaloid toxin cylindrospermopsin (CYN).

Several cyanobacteria species have been reported to synthesize CYN (CYN^+^): *Umezakia natans* (Harada et al. 1994), *A. ovalisporum* (Banker et al. 1997; Shaw et al. 1999), *A. flos-aquae* (Preussel et al. 2006), *Raphidiopsis curvata* (Li et al. 2001) and *R. mediterranea* (McGregor et al. 2011), *Anabaena bergii* (Schembri et al. 2001), *Anabaena lapponica* (Spoof et al. 2006), *Cylindrospermopsis raciborskii* (Hawkins et al. 1985), *Lyngbya wollei* (Seifert et al. 2007), and *Oscillatoria* sp. (Mazmouz et al. 2010). Among these CYN^+^ species, the most abundant and best documented are *C. raciborskii* and *A. ovalisporum*.

CYN^+^ *C. raciborskii* and *A. ovalisporum* species appear to be differentially distributed worldwide. Although, *C. raciborskii*, initially described in tropical zones, is spreading to temperate regions, its CYN^+^ strains have been only found in Australia, Asia, and South America (Sinha et al. 2012). In contrast, CYN^+^ *A. ovalisporum* has been reported in Europe, Middle East, Australia, and North America (Yilmaz et al. 2008; Kinnear 2010). These distribution differences could be due to distinct ecophysiological strategies for the survival of the two species (Everson et al. 2011). Mehnert et al. (2010) and Sukenik et al. (2012) warned about the invasive behavior of *A. ovalisporum* associated with global climate change. Interestingly, except in one case, (Ballot et al. 2011) all strains of *A. ovalisporum* isolated so far are CYN^+^. Moreover, CYN was detected under all culture conditions assayed, including different nutrient settings (Bacsi et al. 2006), temperature, and light intensity (Cires et al. 2011). The presence of two transcriptional start points for *aoaA*-*C* genes was also reported, suggesting the existence of one constitutive promoter (Shalev-Malul et al. 2008). The concentration of CYN detected in blooms dominated by *A. ovalisporum* has usually been higher than that of CYN^+^ *C. raciborskii* blooms, ranging between 9.4 and 18 μg CYN/L (Quesada et al. 2006; Messineo et al. 2010); but larger concentrations up to 120 μg CYN/L in Australia (Shaw et al. 1999) were registered.

A putative CYN biosynthetic pathway has been proposed according to isotope-labeled precursor feeding experiments and genetic data. A partial gene cluster involved in CYN production was first characterized in *A. ovalisporum* (*aoa* genes) (Shalev-Alon et al. 2002; Kellmann et al. 2006), and later the complete gene cluster in *C. raciborskii* (*cyr* genes) (Mihali et al. 2008), *Aphanizomenon* sp. 10E6 (Stüken and Jakobsen 2010), *Oscillatoria* sp. PCC6506 (Mazmouz et al. 2010), and *R. curvata* CHAB1150 (Jiang et al. 2012) were described. Several molecular determinants based on *aoa* (Barón-Sola et al. 2012) and *cyr* (Schembri et al. 2001; Fergusson and Saint 2003; Rasmussen et al. 2008; Ballot et al. 2011) gene sequences, coding for polyketide synthase (PKS), nonribosomal peptide synthetase (NRPS), amidinotransferase (AMDT), or sulfotransferase (*Cyr*J) have been successfully used to discriminate between CYN^+^ and CYN^−^ cyanobacterial strains.

Both *aoa* and *cyr* genes show high similarity, and hypothetically encode, among other proteins, an AMDT, a NRPS, and a PKS. The AMDT was proposed as the first enzyme involved in CYN synthesis (Burgoyne et al. 2000; Kellmann et al. 2006; Mihali et al. 2008). AMDTs catalyze the reversible transfer reaction of an amidino group (donor) to an amine group (acceptor). The characterized AMDTs utilize l-arginine as the main amidino donor substrate, and glycine (Humm et al. 1997b; Lee et al. 2002; Muenchhoff et al. 2010), inosamine phosphate (Fritsche et al. 1998), or lysine (Hernandez-Guzman and Alvarez-Morales 2001) as acceptor molecules. In general, AMDTs can use a wide variety of substrates. An exception is the AMDT from *C. raciborskii* AWT205, CyrA, the only cyanobacterial AMDT characterized to date (Muenchhoff et al. 2010, 2012). In effect, CyrA, encoded by the *cyrA* gene, can use only l-arginine as a donor of the amidino group, and glycine as acceptor; therefore, it is considered an l-arginine:glycine AMDT (Muenchhoff et al. 2010).

The important contribution of *A. ovalisporun* to CYN production due to its ability to constitutively synthesize the toxin at high level, its worldwide distribution and invasive trend, and the scarce information about CYN synthesis and its regulation, led us to study the protein encoded by the *aoaA* gene, a putative AMDT. The *aoaA* was cloned, the protein product (AoaA) overexpressed, purified and characterized biochemically, confirming its AMDT activity. Initial studies on the control of CYN synthesis by AoaA and the modulation of the AMDT activity by temperature, pH, and some cations were performed. In addition, AoaA was phylogenetically compared with other AMDTs of diverse origin.

## Materials and Methods

### Strains and vectors

The strain *A. ovalisporum* UAM-MAO (Barón-Sola et al. 2012) was isolated from a Spanish pond of the Parque Juan Carlos I in Madrid (40° 27′ N; 3° 36′ W), used for various recreation activities. The mean temperature and pH in the water at the time of sampling was 22°C and 7.2, respectively. The strain was identified according to Komarek and Anagnostidis (1989), and cultured in BG11 medium under continuous white light (50 μmol m^-2^ S^-1^) at 28°C.

Two *Escherichia coli* strains were utilized: DH5α, for cloning and sequencing the *aoa*A gene into pET28b+ vector (Novagen, Madison, WI), and BL21 (DE3) used for protein overexpression.

### Cloning of aoaA and overexpression of AoaA

Cyanobacteria DNA extraction and purification were carried out following the method developed by Smoker and Barnum (1988), and modified by Neilan (1995). The *aoa*A gene was amplified by polymerase chain reaction (PCR) with the specific primers *aoa*A-F (5′-AAAAGAATTCGATGCAAACAGGAATTGTAAATAGCTG-3′) and *aoa*A-R (5′-AAAAAAGCTTCAAACCTACTAAATAATGATGAAGCG-3′), containing EcoRI and HindIII restriction sites, respectively. The *aoa*A sequence described by Shalev-Alon et al. (2002) was used as template to design the primers. The PCR product and pET28b+ vector were double digested using EcoRI and HindIII enzymes (Takara Bio Inc., Sigha, Japan) and purified from agarose gel using GFX PCR DNA Gel Band Purification Kit (GE Healthcare, Waukesha, WI) for subsequent ligation. The constructed vector was first transformed into *E. coli* DH5α for sequence analysis and also introduced into *E. coli* BL21 (DE3) for expression as an N-terminal His_6_-tagged fusion protein. Plasmid purification was performed with Zyppy™ Plasmid Miniprep Kit (Zymo Research Cia., Invine, CA). Expression of the recombinant His_6_-tagged *aoa*A was performed in Luria Bertani (LB) medium supplemented with 30 μg/L of kanamycin and 0.1 mmol/L isopropyl β-d-1-thiogalactopyranoside (IPTG) at 28°C and constant agitation (200 rpm).

### Purification of AoaA

Cells of the recombinant suspension culture were harvested by centrifugation (6000*g* for 15 min at 4°C). The pellet was suspended in chilled lysis buffer (50 mmol/L HEPES, pH 7.5, 500 mmol/L NaCl, 5% glycerol, 0.5 mmol/L dithiothreitol, 0.5 mmol/L phenylmethanesulfonyl fluoride, 30 mmol/L imidazole). Cell lysis was performed by sonication for 10 min in an ice bath with a Braun Labsonic 2000, using a 100 W needle probe. The crude extract was centrifuged 12000*g* for 15 min, and the supernatant loaded onto a HiTrap Chelating Column (GE Healthcare, Waukesha, WI) for protein purification, according to manufacturer instructions. An imidazole gradient (100–500 mmol/L) was used for protein elution, and the eluted fractions were subjected to SDS-PAGE (sodium dodecyl sulfate polyacrylamide gel electrophoresis) followed by Coomassie blue staining. The fractions containing the purified recombinant protein AoaA were pooled and desalted with an Amicon filtration unit 10 kDa (Amicon® Ultra, Millipore, Billerica, MA). The buffer (50 mmol/L HEPES, pH 7.5, 10% glycerol, 10 mmol/L dithiothreitol) was used for protein dilution and storage at −80°C. Protein concentration was determined by the Bradford (1976) method using bovine serum albumin as a protein standard.

### Protein sequence analysis

**Figure 1:**
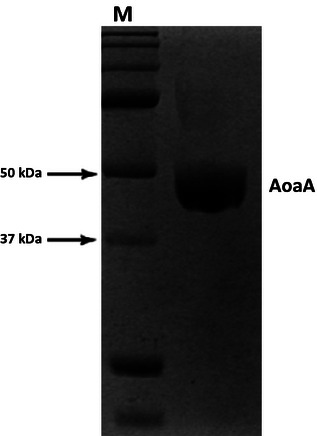
SDS-polyacrylamide gel electrophoresis of purified AoaA from *Aphanizomenon ovalisporum* UAM-MAO. M, molecular weight marker lane. The recombinant AoaA was expressed in *Escherichia coli* BL21 (DE3), and contained an N-terminal 6-His tag and extra-encoded vector amino acids.

The protein band corresponding to 50.2 kDa on SDS-PAGE was excised (Fig. 1), destained, and extracted. The recombinant protein AoaA was digested with trypsin, and the resulting peptides analyzed by matrix-assisted laser desorption/ionization time-of-flight mass spectrometry. The AoaA amino acid sequence obtained was submitted to BLAST (GenBank accession number AEQ64884) for sequence comparison. Clone Manager software was used to calculate the predicted molecular mass and theoretical isoelectric point (Ip). Sequence alignments were performed by MEGA5 software (Tamura et al. 2011).

### Phylogenetic analysis

Twenty-nine AMDT amino acid sequences from the GenBank database were aligned, using the ClustalW tool from MEGA5 software (Tamura et al. 2011). A neighbor-joining phylogenetic tree (1000 bootstrap) was constructed, following the substitution model of Jones–Taylor–Thornton (Jones et al. 1992).

### Assay of AMDT activity

The AMDT activity was determined colorimetrically by measuring the production of ornithine with ninhydrin reaction at low pH (Van Pilsum et al. 1970), following the modifications of Muenchhoff et al.(2010). Briefly, unless specified, the reaction mixture contained in a final volume of 300 μL: 50 mmol/L Tris-HCl pH 8.5, 20 mmol/L l-arginine and 20 mmol/L glycine as substrates, and 8–12 μg of purified AoaA. The reactions were performed in triplicate at 30°C during 30 min. The specific AMDT activity is expressed as micromole ornithine formed per minute and per microgram of protein.

### Identification of the products of the AMDT activity

The AMDT reaction of AoaA was carried out in a final volume of 4 mL, containing 20 mmol/L l-arginine and/or glycine. After incubating for 3 h at 30°C, AoaA was removed using a 10 kDa Amicon filtration unit (Amicon® Ultra). The filtrate and standard compounds (Sigma-Aldrich™ Inc., St. Louis, MO) were analyzed by LC ESI-MS (Liquid Chromatography-Electrospray Ionization Mass Spectrometry). The chromatographic separation was carried out by high-performance liquid chromatography (model 1100 Agilent Technologies, Santa Clara, CA), using the Zorbax C18 XDB 5 μm (50 × 2.1 mm) column and a mobile phase gradient. The mobile phase consisted of two eluents: (A) water with 0.1% acetic acid; and (B) acetonitrile with 0.1% acetic acid. The continuous gradient performed during 15 min was: *t*_0_, A = 95%; *t*_15_, A = 5%. The flow rate was 0.5 mL/min, and the injection volume 5 μL. The ESI-MS analyzer was a Q-TOF QSTAR AB SCIEX (Quadrupole/Time-Of-Flight (Q-TOF) tandem mass spectrometer, model QSTAR AB SCIEX) operated in the positive ionization mode, under the following conditions: ion spray voltage, 5.5 kV; ion source gas pressure, 1 and 2.5 psi; two declustering potentials, 3 and 15 V; and focusing potential, 210 V.

### Kinetic analysis of the AMDT reaction

The kinetic parameters of the AMDT reaction were obtained by nonlinear regression analyses with the enzyme kinetics module of GraphPad prism 5.03. All kinetic analyses were repeated at least three times with reproducible results.

### Assay of AMDT inhibition by guanidine acetate

AoaA activity was measured by adding guanidine acetate (GAA) at different concentrations (1–30 mmol/L) to the AMDT reaction mixtures that included varied concentration of glycine (0, 1, 3, 6, 15, and 20 mmol/L). In all cases, 30 mmol/L arginine was used. The reactions were performed in triplicate. Other conditions were as stated above for AMDT assay (2.6).

### Assay of AMDT activity and stability of AoaA at different pH and temperature

The AMDT activity of purified AoaA was determined at 30°C in 100 mmol/L buffers of pH 5.5–10 (pH 5.5–6.5, Mes-NaOH; pH 7–7.4, K_2_HPO_4_-KH_2_PO_4_; pH 8–9, Tris-HCl; and pH 9.5–10, Ches-NaOH). The pH stability of AoaA was evaluated by preincubating AoaA at 30°C with the different buffers during 1 h, and measuring the AMDT activity every 15 min.

The effect of temperature on AMDT activity of AoaA was determined by measuring the amount of ornithine formed at pH 8.5 at various temperatures, ranging from 15 to 50°C. The temperature stability was analyzed by preincubating AoaA at pH 8.5 and at 15–45°C during 1 h, and measuring the AMDT activity every 10 min.

### Assay of cation influence on AMDT activity of AoaA

To assess the effect of metal ions on AoaA activity, various divalent cations (Ca^2+^, Mg^2+^, Mn^2+^, Co^2+^, Fe^2+^, Ni^2+^) were added to the AMDT reaction mixture at different concentrations (0.01, 0.1, 1, and 5 mmol/L). At the end of the reaction, ethylenediaminetetraacetic acid (EDTA) was added to a final concentration of 0.1 mmol/L, and the reaction mixture was centrifuged (12000*g* for 10 min). The supernatant was used to measure the ornithine formed during the reaction. All assays were performed in triplicate.

## Results

### Gene expression and purification of the recombinant protein AoaA of *A. ovalisporum*

The recombinant AoaA protein was purified, reaching more than 95% purity, as shown by SDS-PAGE (Fig. 1). The mean yield of the purified AoaA was 11.2 mg/L of culture. The molecular mass predicted from the gene sequence was 45.7 kDa, and the calculated Ip was 5.79. In the SDS-PAGE gel (Fig. 1), the molecular mass appeared slightly larger (50.2 kDa), due to the additional six His residues and the extra encoded amino acids from the vector.

**Figure 2:**
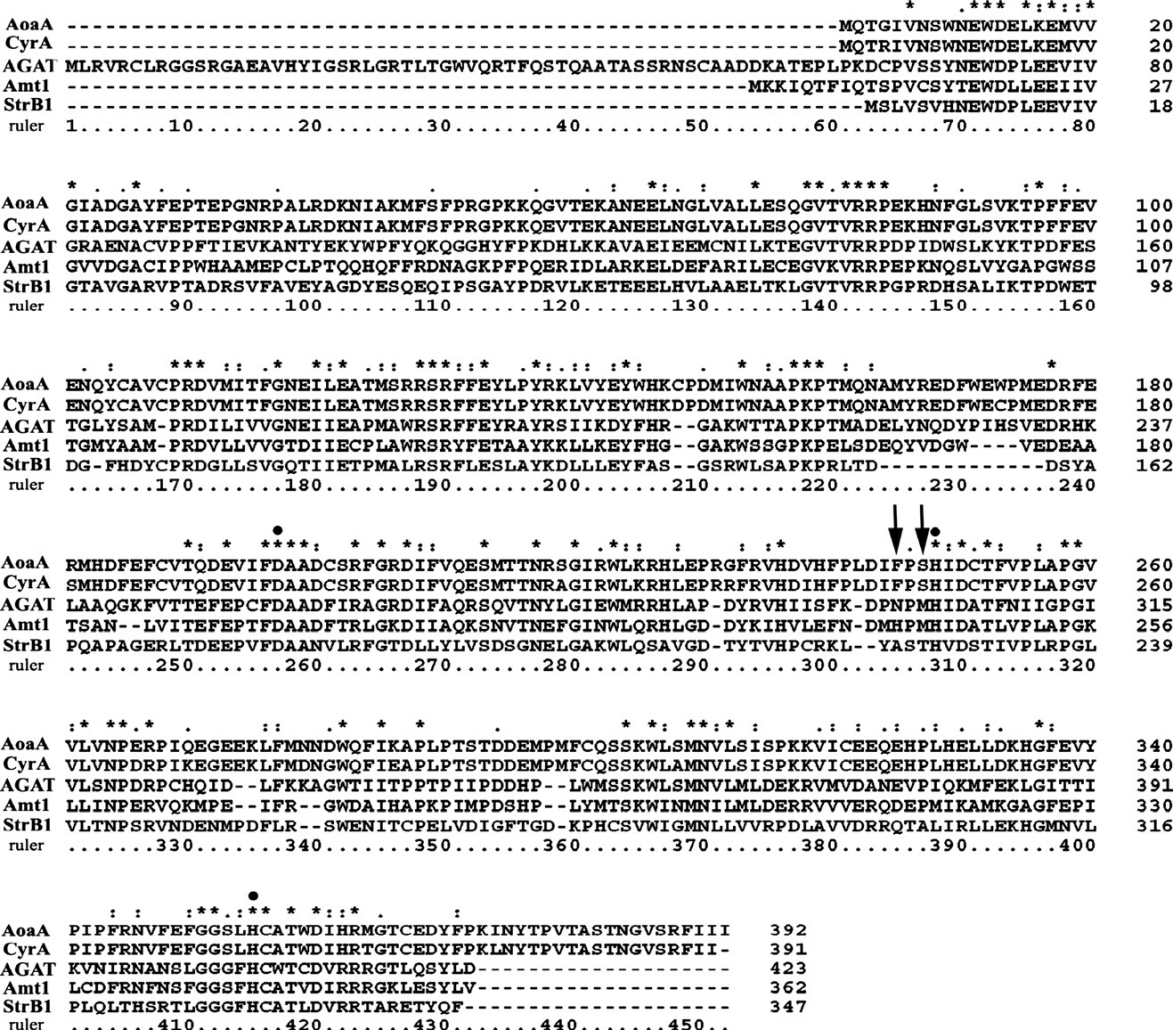
Alignment of amidinotransferase (AMDT) amino acid sequences obtained from databases. Fully conserved amino acids (asterisk). Residues with both conserved size and hydropathy (dot). Residues with either conserved size or hydropathy (two dots). Conserved amino acid within the catalytic site (arrows). Only AoaA and CyrA conserved amino acid in the active site (asterisk plus dot). AoaA, *Aphanizomenon ovalisporum* UAM-MAO; CyrA, *Cylindrospermopsis raciborskii* AWT205; AGAT, *Homo sapiens*; Amt1, *Pseudomonas syringae* pv. *phaseolicola*; StrB1, *Streptomyces griseus*.

The recombinant purified AoaA was sequenced by MS fingerprinting after trypsin digestion. More than 90% of the sequence was obtained, confirming a nontruncated form of the expected protein, a putative AMDT. Alignment of AoaA, CyrA, human AGAT, Amt1 (from *Pseudomonas syringae*), and StrB1 (from *Streptomyces griseus*) sequences showed good homology among these proteins, especially between AoaA and CyrA, with approximately 96% identity (Fig. 2). The three amino acids related to the catalytic site of human AGAT (Humm et al. 1997a), Asp^254^, His^303^, and Cys^407^, are conserved in AoaA (Asp^197^, His^248^, and Cys^356^). As in CyrA (Muenchhoff et al. 2010), two residues of the human AGAT catalytic site, Asn^300^ and Met^302^, are substituted by Phe^245^ and Ser^247^, respectively. Nevertheless, several amino acids are not conserved in the AoaA sequence.

### Phylogenetic analysis

**Figure 3:**
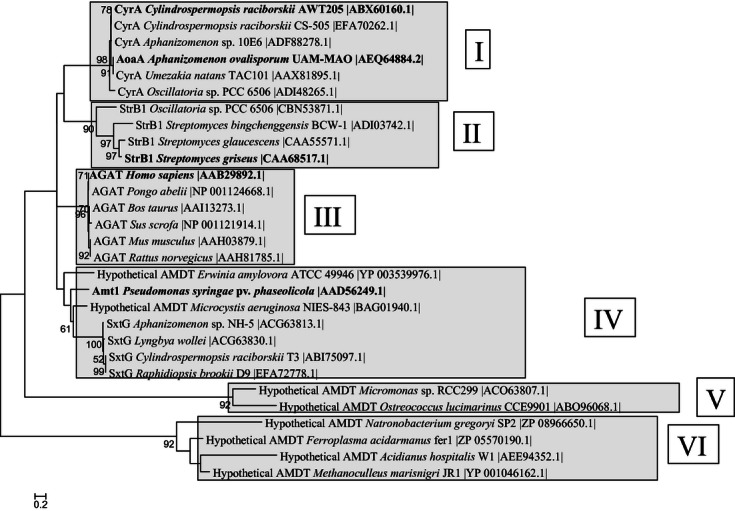
Dendrogram of amidinotransferase (AMDT) amino acid sequences. Twenty-eight sequences from the GenBank database, besides that of the AoaA product were used. The neighbor-joining method was applied with a bootstrap value of 1000, following the substitution model of Jones–Taylor–Thornton. Clusters are indicated by roman numbers. In bold, sequences from already characterized proteins.

To explore the phylogenetic relationship of AoaA with other AMDTs, its amino acid sequence was compared with those of 28 AMDTs from different origins found in Genbank database (Fig. 3). Most of the proteins referred to putative AMDTs; only in five instances the AMDT activity has been proven. The resulting phylogenetic tree (Fig. 3) comprises six clusters. Cluster I contains only AMDTs from CYN^+^ species, AoaA, and CyrA being integrated in this group. Cluster II includes AMDT sequences from *Streptomyces* species (StrB1) together with one protein from the cyanobacterium *Oscillatoria* sp PCC 6506, which presents two types of AMDTs. Cluster III groups animal AMDTs, including the well-studied human AGAT. Cluster IV collects protein sequences from saxitoxin-producing cyanobacteria from diverse origins and two hypothetical AMDTs. Clusters V and VI are quite separated from the previous clusters. Cluster V comprises putative AMDTs from green algae species. Cluster VI, rather distant from the others, incorporates putative AMDTs from Archae domain.

The phylogenetic tree shows the close relationship not only between the recombinant purified AMDTs from *C. raciborskii* AWT205 (CyrA) and *A. ovalisporum* UAM-MAO (AoaA), but also among putative AMDTs from other CYN^+^ cyanobacteria: *C. raciborskii* CS-505, *Aphanizomenon* sp. 10E6, *Oscillatoria* sp. PCC6506, and *Umezakia natans* TAC101. However, another AMDT from *Oscillatoria* sp. PCC6506 is also close to that of *Streptomyces* (cluster II), and clearly appears separated from the CYN^+^ cyanobacteria group (cluster I).

### Analysis of AMDT activity

**Figure 4:**
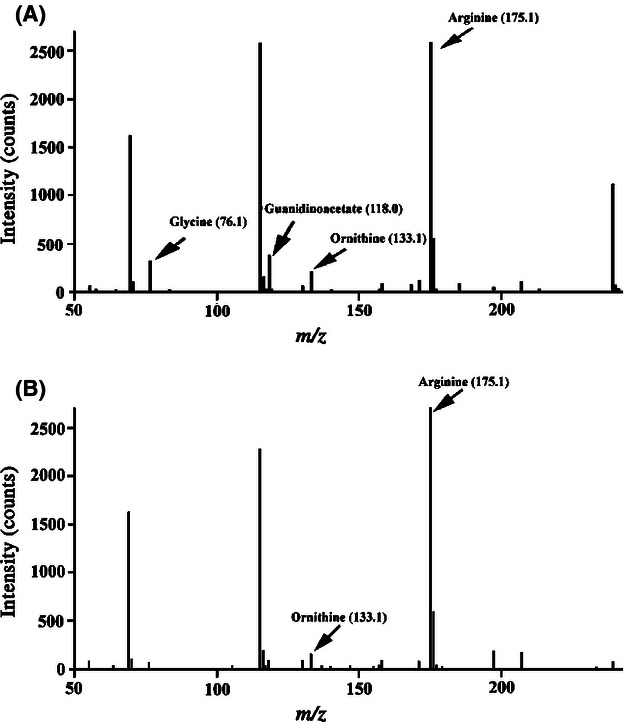
ESI-MS spectra of substrates and products in the amidinotransferase (AMDT) reaction of AoaA. (A) l-arginine (175.1) and glycine (76.0) were used as substrates. Ornithine (133.1) and guanidinoacetate (118.0) were the reaction products. (B) l-arginine (175.1) was used as unique substrate. Ornithine (133.1) was the reaction product.

Considering the great similarity of the amino acid sequence in AoaA and CyrA, and knowing that CyrA was an AMDT, it seemed obvious to confirm that AoaA was also an AMDT. The recombinant purified AoaA showed AMDT activity, as revealed by the formation of ornithine and GAA when it was incubated with l-arginine and glycine (Fig. 4A). However, GAA was not detected when glycine was omitted in the reaction mixture (Fig. 4B), strongly indicating that both glycine and l-arginine were acting as AMDT substrates, and suggesting a ping-pong mechanism in the enzymatic activity.

**Figure 5:**
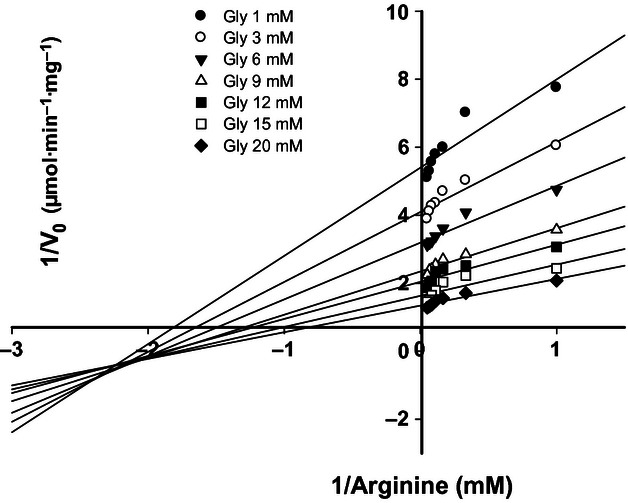
Amidinotransferase (AMDT) kinetics of the AMDT reaction of AoaA. Double reciprocal plot of the initial enzyme velocity versus the l-arginine concentration at the glycine concentrations indicated in the graph insert.

At constant concentrations of l-arginine and glycine (20 mmol/L each), the AMDT activity of AoaA was linear over a time period of 45 min. Linearity was also observed with respect to enzyme quantity between 2.5 and 20 μg. The kinetic parameters of AoaA AMDT found at 30°C and pH 8.5 were: *V*_max_, 0.62; *K*_mArg_ and *K*_mGly,_ 0.74 ± 0.2 and 5.68 ± 0.7, respectively (Table 1). In the kinetics representation of Fig. 5, the lines intersect at the left of the *y*-axis and below the *x*-axis indicating that binding of one substrate to the enzyme diminishes the affinity for the other substrate; therefore, AoaA seems to bind to glycine and l-arginine in a random way before releasing the first reaction product, suggesting a random sequential mechanism. However, ornithine was also produced when only l-arginine was present (Fig. 4B). This result would not support a typical random sequential mechanism. Thus, taken together the data of Figures 4 and 5 suggest a hybrid random sequential ping-pong mechanism for the AMDT activity of AoaA.

**Table 1:** Some kinetic and chemical features of characterized amidinotransferases

	AoaA^1^	CyrA^2^	AGAT^3^	Gm AMDT^4^
*V*_max_ (μmol/min per·mg)	0.62	1.05	0.44	ND
*K*_m_ l-arginine (mmol/L)	0.74 ± 0.2	3.5 ± 1.14	2 ± 0.5	3.8
*K*_m_ glycine (mmol/L)	5.8 ± 0.7	6.9 ± 2.7	3 ± 1	0.89
Optimal temperature (°C)	32–37	32	37	37
Optimal pH	8.5–9	8.5	7.5	9.5

AoaA, *Aphanizomenon ovalisporum* UAM-MAO; CyrA, *Cylindrospermopsis raciborskii* AWT205; AGAT, *Homo sapiens*; Gm, *Glycine max*; AMDT, amidinotransferase.

1 This study.

2 Muenchhoff et al. (2010).

3 Fritsche et al. (1997).

4 Lee et al. (2002).

**Figure 6:**
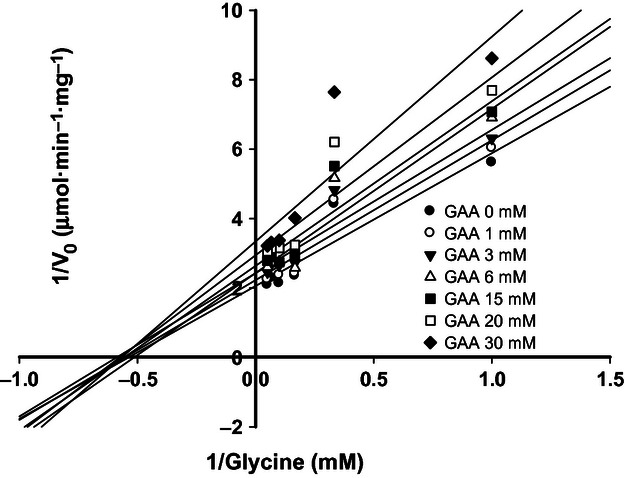
Effect of guanidino acetate on amidinotransferase (AMDT) reaction of AoaA. Double reciprocal plot of the initial velocity versus l-glycine concentration at the guanidine acetate (GAA) concentrations indicated in the graph insert. l-arginine was maintained at saturated concentration (30 mmol/L).

When GAA was included at different concentrations in the AMDT reaction mixture using various glycine concentrations, the double reciprocal plot representating the group of the resulting lines intersect on the *x*-axis (Fig. 6), indicating a noncompetitive inhibition effect by GAA of the AoaA AMDT activity.

### Influence of diverse factors on AoaA activity and stability

The formation of cyanobacterial blooms depends on physical and chemical environmental changes; therefore, CYN production would also probably be influenced by those changes. On this basis, we thought it would be interesting to evaluate the AMDT activity and stability of AoaA at different pH, temperature, and cation concentration.

**Figure 7:**
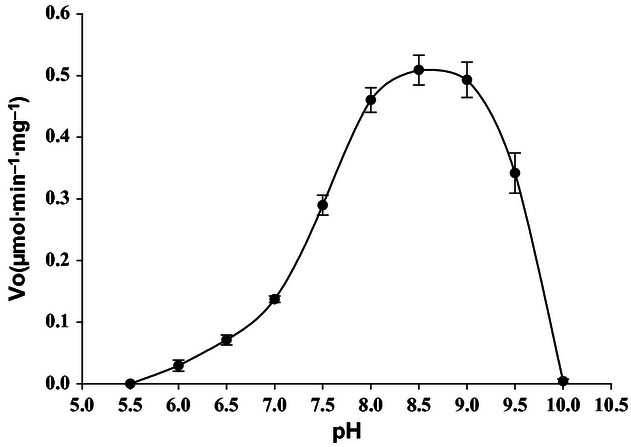
Effect of pH on amidinotransferase (AMDT) activity of AoaA. The assay was performed at 30°C during 30 min in the pH range 5.5–10. Data are given as means (*n* = 3), and error bars represent SD.

**Figure 8:**
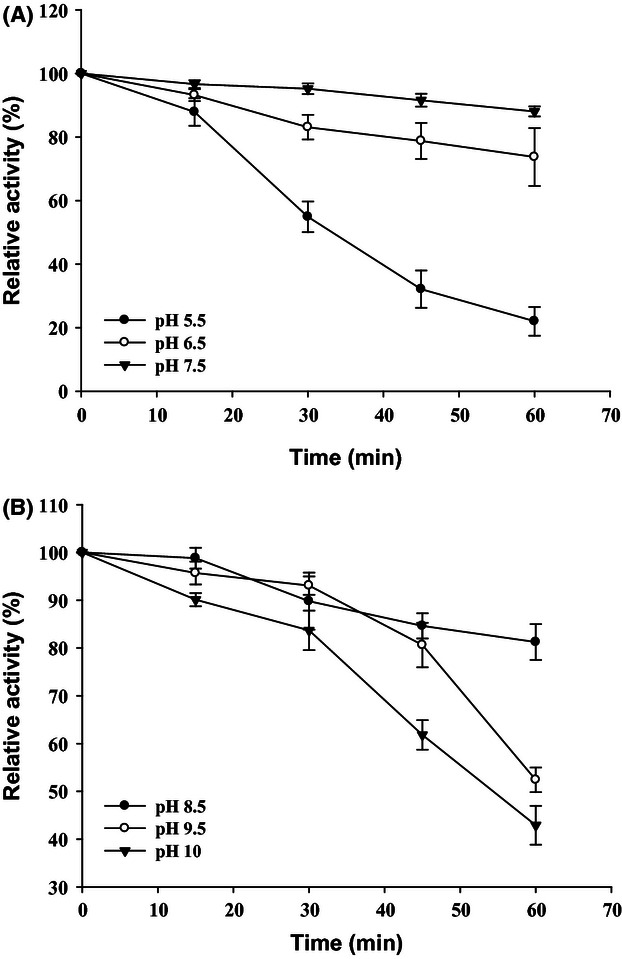
Characterization of AoaA stability under different pH. (A), pH 5–7.5; (B), pH 8.5–10. Purified AoaA was preincubated at 30°C and several pH buffers for varying time periods (0–60 min) and then assayed for residual activity. Data are given as means (*n* = 3), and error bars represent SD.

The AMDT activity and stability of the recombinant purified AoaA were assayed in the pH range 5.5–10, and it was found that both were not significantly affected by usual pH values in water ecosystems. Although the highest activity was attained between 8.5 and 9 (Table 1), almost 60% of the activity remained at pH 7.5 and 9.5 (Fig. 7). The maximum stability, tested by AMDT activity after preincubation of AoaA for 1 h at 30°C, was found at pH 7.5 (Fig. 8A); but more than 50% of the initial activity was retained at 6.5 and 9.5 (Figs. 7B and 8A).

**Figure 9:**
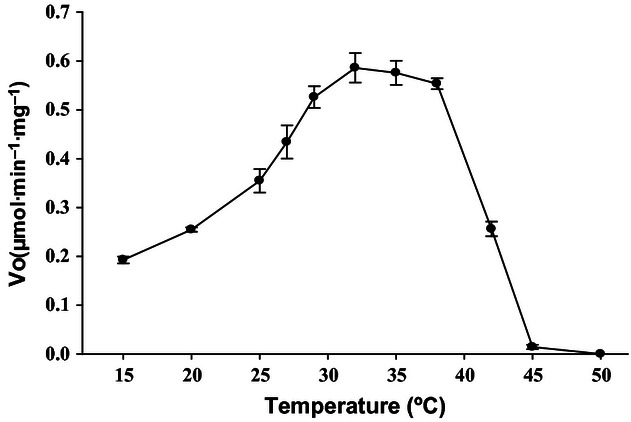
Effect of temperature on amidinotransferase (AMDT) activity of AoaA. The assay was performed with Tris-HCl pH 8.5, during 30 min at the temperature range 15–45°C. Data are given as means (*n* = 3), and error bars represent SD.

**Figure 10:**
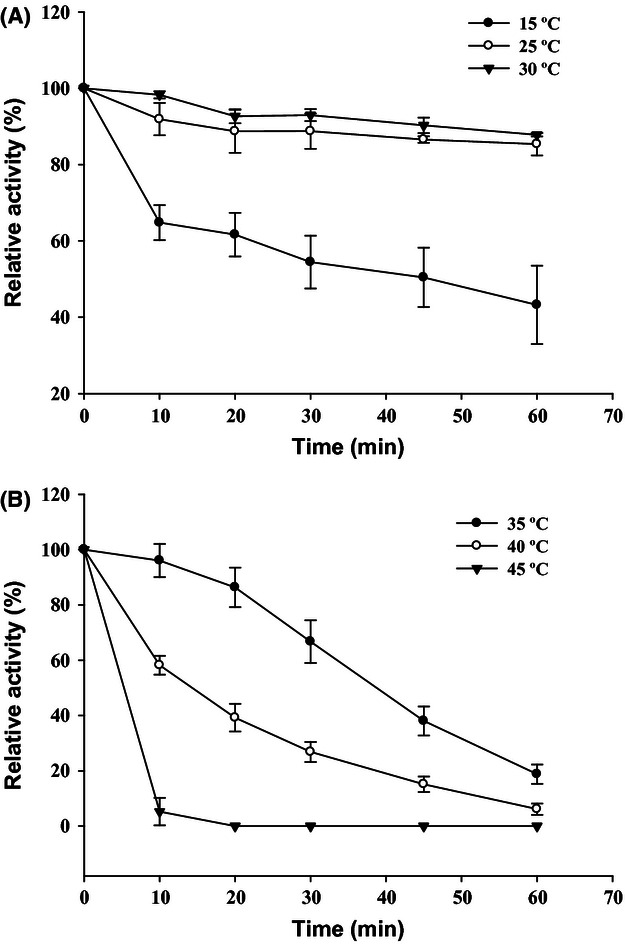
Characterization of AoaA stability at different temperatures: (A) 15–30°C; (B) 35–45°C. Purified AoaA was preincubated in Tris-HCl pH 8.5, at different temperatures for varying time periods (0–60 min), and then assayed for residual activity. Data are given as means (*n* = 3), and error bars represent SD.

Thermal activity was tested between 15°C and 50°C. Special care was taken to perform the assays of temperature and pH within the time range in which the activity was linear, to prevent side effects of AoaA stability. Figure 9 shows that although the optimal temperature was at 32°C, subtle differences were found between 30°C and 37°C (Table 1) with more than 70% activity remaining between 25°C and 30°C. In relation to thermal inactivation, the maximum stability was found between 25°C and 30°C, as at both temperatures more than 90% activity remained after 60 min (Fig. 10A). At 40°C, 75% of activity was lost after 30 min, and at 45°C, AoaA was completely inactivated after 10 min (Fig. 10B).

**Figure 11:**
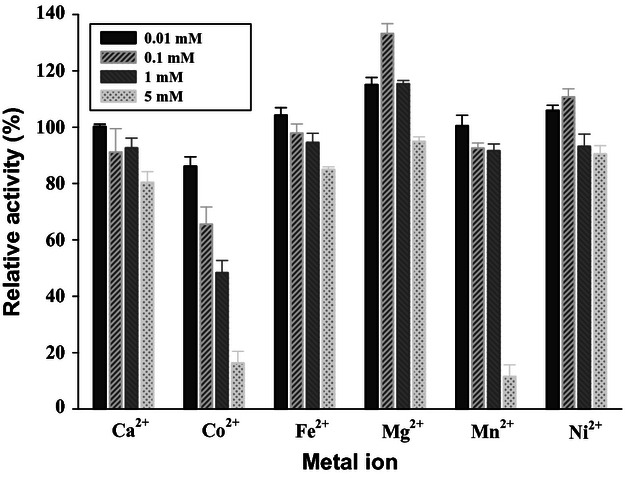
Effect of divalent cations on amidinotransferase (AMDT) activity of AoaA. Enzyme activity was measured in the presence of different concentrations (0.01–5 mmol/L) of the divalent cations indicated in the graph during 30 min. Data are given as means (*n* = 3) and error bars represent SD. The 100% activity level was 0.47 μmol/min per mg.

Fluctuations of ion concentration in dynamic water systems are usual events. On the other hand, divalent cations intervene in numerous enzymatic activities, being able to modulate them in a direct or indirect way. For those reasons, the AMDT activity of AoaA was determined in the presence of various divalent cations at different concentrations (0.01, 0.1, 1, and 5 mmol/L). The cations tested were Ca^2+^, Co^2+^, Fe^2+^, Mn^2+^, and Ni^2+^. Fe^2+^, Ni^2+^, and Ca^2+^ did not affect significantly the AMDT activity; however, Co^2+^ at all concentrations assayed produce inhibition, and Mn^2+^ at 5 mmol/L inhibited the activity 80% (Fig. 11). Curiously, Mg^2+^ at 0.1 mmol/L showed a clear stimulatory effect, enhancing the activity by around 30%.

## Discussion

Cyanobacterial AMDTs are involved in the synthesis of CYN and saxitoxin, but until now only the AMDT from the CYN producer *C. raciborskii* AWT205, CyrA, has been characterized (Muenchhoff et al. 2010). To widen the understanding of CYN synthesis, the *aoaA* gene of the CYN^+^ *A. ovalisporum* strain UAM-MAO, isolated from a Spanish artificial lake, was cloned, overexpressed, and the recombinant protein, AoaA, characterized.

AoaA appears to be very similar to CyrA. In both proteins, the amino acid sequence is almost identical, and two residues of the human AGAT catalytic site, Asn^300^ and Met^302^, are substituted by Phe^245^ and Ser^247^, respectively (Fig. 2). Recently, it has been stated (Muenchhoff et al. 2012) the role of these two residues in CyrA with the narrow substrate specificity previously observed by Muenchhoff et al.(2010).

Phylogenetically, AoaA is closely related not only to CyrA but to other putative AMDTs of different CYN^+^ cyanobacteria (Fig. 3). The phylogenetic analysis shows other interesting data. For example, the position of one of the hypothetical *Oscillatoria* sp PCC6506 AMDTs appears to be closer to *Streptomyces* AMDT than to those of CYN-producing cyanobacteria. Interestingly, this *Oscillatoria* AMDT might use inosamine phosphate as amidino acceptor instead of glycine, as it is the case of AMDTs of the CYN producers. It is also worthwhile to mention the great phylogenetic proximity of AoaA to vertebrate AMDTs (Fig. 3).

AoaA, like CyrA, can use both glycine and arginine as substrates, giving rise to ornithine and GAA as reaction products (Fig. 4). The *V*_max_ and *K*_m_ for glycine of the two AMDTs is similar; but the affinity for arginine is higher in AoaA (Table 1). For more than a decade, glycine has been considered as a substrate in CYN synthesis, as in experiments with isotope-labeled glycine it was shown that this amino acid was incorporated in the CYN molecule (Burgoyne et al. 2000). However, in the same experiments l-arginine was not incorporated in the toxin, suggesting that it might not be an adequate substrate in CYN synthesis. Our data (Fig. 4A) along with those of Muenchhoff et al. (2010) seem to contradict that suggestion, as l-arginine is clearly a substrate in the AMDT reaction catalyzed by AoaA (Fig. 4A).

The likeness between AoaA and CyrA also applies to the enzymatic mechanism involved in the AMDT reaction, a mixed ping-pong random sequential system. The hybrid system in AMDT of AoaA can be taken from the data of the double reciprocal plot of the activity with l-arginine as the varied substrate (Fig. 5), along with the analysis of the reaction products, ornithine or GAA, found in the presence of either, l-arginine plus glycine (Fig. 4A) or solely l-arginine (Fig. 4B), respectively. Therefore, the mixed kinetic system of AoaA and CyrA observed with glycine and l-arginine would differentiate these cyanobacterial AMDTs from AGAT (Fritsche et al. 1997), which exhibits a clear ping-pong mechanism.

We have also observed a noncompetitive inhibition of AoaA by GAA using glycine as a varied substrate, in support of a sequential mechanism (Fig. 6). GAA might bind to AoaA out of the catalytic center inducing conformational changes that would decrease AoaA activity. Therefore, GAA could also be considered as a product inhibitor and regulator of AMDT activity, as other previously described compounds produced in different metabolic pathways such as ornithine (Sipila 1980; Muenchhoff et al. 2010), creatine (Guthmiller et al. 1994), putrescine, or spermidine (Lee et al. 2002). Ornithine was identified as a strong inhibitor of CyrA activity (Muenchhoff et al. 2010) and rat AMDT (Sipila 1980), but the type of inhibition seems to be different from that observed with GAA in AoaA (Fig. 5). Ornithine caused a partial mixed inhibition of CyrA (Muenchhoff et al. 2010) and a competitive inhibition of vertebrate AMDT (Sipila 1980). It would be worthwhile to assay if GAA inhibits CyrA and vertebrate AMDT activity, and if ornithine inhibits AoaA activity.

The optimal temperatures for maximal activity (*T*_max_) and the highest stability of AoaA were between 32–37°C (Fig. 8) and 25–30°C, respectively (Fig. 9A). The pHs for maximal activity and highest stability were between 8.0–9.0 and 7.5, respectively. Considering the mean temperature and pH registered in the water body from which the *A. ovalisporum* UAM-MAO under study was isolated (22°C and pH 7.2), it appears that AoaA was acting under suboptimal conditions; yet, the protein would be stable, a fact that could compensate the low activity in CYN production. It would be interesting to study whether there are differences between the optimal temperature and pH for activity and stability of AoaA in *A. ovalisporum* strains isolated from other ecosystems.

In general, the temperature and pH values for maximal activity and stability obtained with AoaA differed from those with CyrA, as in CyrA the activity appeared to be optimal at 32°C and pH 8.5, and the highest stability at pH 6.5. The origin of those differences might be attributed to the distinct amino acids outside the active sites in the two proteins (Fig. 2).

AoaA activity was affected by divalent cations, normally present in water habitats and the majority utilized by cyanobacteria for structural or catalytic purposes. Among the six cations assayed, Ca^2+^, Co^2+^, Fe^2+^, Mg^2+^, Mn^2+^, and Ni^2+^, the most remarkable effects were observed with Mg^2+^ and Co^2+^. Mg^2+^ enhanced the activity at the lower concentrations used (0.01–1 mmol/L) and had no effect at the highest concentration (0.5 mmol/L). Co^2+^ at all concentrations tested (0.01–5 mmol/L) inhibited AoaA activity, the inhibition being 30% at 0.1 mmol/L and 50% at 1 mmol/L (Fig. 11). The highest concentration (5 mmol/L) of Mn^2+^ and Ca^2+^ was inhibitory. Few data are available on the effect of metal ions on purified AMDTs. While *Glycine max* AMDT activity was not affected by monovalent and divalent cations (Lee et al. 2002), recombinant AGAT enzyme was strongly inhibited by Hg^2+^ (1 mmol/L), Zn^2+^ (1 mmol/L), and Ni^2+^ (10 mmol/L) (Humm et al. 1997b). As in our experiments the cations were only present during the AMDT reaction assay, it seems reasonable to think that the effect observed is direct on AoaA and does not result from acting on other enzyme activities involved in cyanobacteria metabolism that could in the end affect AoaA. Several questions arise from the cation data of Fig. 11, including: (i) how Mg^2+^ and Co^2+^ can act on AoaA activity?; (ii) are the intracellular concentration of these two ions enough to justify the effects observed?; and (iii) could the different cations exhibit a synergistic or antagonic effect? Our results do not allow drawing any conclusion on the three posed questions. However, it could be hypothesized that Mg^2+^ and Co^2+^ effects are related to the dimer or tetramer structure conformation that active AMDTs could adopt (Humm et al. 1997a; Muenchhoff et al. 2010). On the other hand, the intracellular Mg^2+^ concentrations that would be required to enhance the AMDT activity of AoaA appear to be normal in cyanobacteria. In effect, Mg^2+^ not bound to chlorophyll has been reported to accumulate in cyanobacterial cells at concentrations in the mmol/L range (Utkilen 1982). Such concentrations are of the same magnitude of those found for Ca^2+^ (Torrecilla et al. 2000). As far as we know, no data are available on intracellular Co^2+^ concentration, in spite of this cation being part of important proteins, such as cobalamins. In general, few data have been reported on intracellular concentrations of ions in cyanobacteria and on the homeostatic mechanisms to maintain them. With respect to the possible interaction among the different cations tested, future experiments should be performed to assess this possibility, as the resulting data could be important in the study of the regulation of AMDT activity in CYN synthesis.
